# A New Score to Predict the Resectability of Pancreatic Adenocarcinoma: The BACAP Score

**DOI:** 10.3390/cancers12040783

**Published:** 2020-03-25

**Authors:** Charlotte Maulat, Cindy Canivet, Célia Touraine, Sophie Gourgou, Bertrand Napoleon, Laurent Palazzo, Nicolas Flori, Guillaume Piessen, Pierre Guibert, Stéphanie Truant, Eric Assenat, Louis Buscail, Barbara Bournet, Fabrice Muscari, the BACAP Consortium

**Affiliations:** 1 The Digestive Surgery and Liver Transplantation Department, Toulouse University Hospital, 31400 Toulouse, France; muscari.f@chu-toulouse.fr; 2 INSERM U1037, The Toulouse Cancer Research Center, Toulouse University, 31100 Toulouse, France; buscail.l@chu-toulouse.fr (L.B.); bournet.b@chu-toulouse.fr (B.B.); 3 The Gastroenterology and Pancreatology Department, Toulouse University Hospital, 31400 Toulouse, France; canivet.c@chu-toulouse.fr; 4 Biometrics Unit, Montpellier Cancer Institute, University of Montpellier, 34000 Montpellier, France; celia.touraine@icm.unicancer.fr (C.T.); sophie.gourgou@icm.unicancer.fr (S.G.); 5 The Jean Mermoz private hospital, Ramsay Général de Santé, 69008 Lyon, France; bertrand.napoleon@dartybox.com; 6 Trocadero Clinic, 75116 Paris, France; laurent.palazzo@wanadoo.fr; 7 The Gastroenterology Department, Montpellier Cancer Institute, University of Montpellier, 34000 Montpellier, France; nicolas.flori@montpellier.unicancer.fr; 8 Department of Digestive and Oncological Surgery, Lille University Hospital, 59000 Lille, France; guillaume.piessen@chru-lille.fr; 9 CANTHER laboratory “Cancer Heterogeneity, Plasticity and Resistance to Therapies” UMR-S1277 INSERM, Team “Mucins, Cancer and Drug Resistance”, 59000 Lille, France; 10 The Léon Bérard Center, 69008 Lyon, France; pierre.guibert@lyon.unicancer.fr; 11 Department of Digestive Surgery and Transplantations, Lille University Hospital, 59000 Lille, France; stephanie.truant@chru-lille.fr; 12 The Saint Eloi Hospital, University Hospital, 34000 Montpellier, France; e-assenat@chu-montpellier.fr

**Keywords:** pancreatic ductal adenocarcinoma, pancreatic cancer, predictive factors, resectability, locally advanced, metastasis, BACAP cohort, score

## Abstract

Surgery remains the only curative treatment for pancreatic ductal adenocarcinoma (PDAC). Therefore, a predictive score for resectability on diagnosis is needed. A total of 814 patients were included between 2014 and 2017 from 15 centers included in the BACAP (the national Anatomo-Clinical Database on Pancreatic Adenocarcinoma) prospective cohort. Three groups were defined: resectable (Res), locally advanced (LA), and metastatic (Met). Variables were analyzed and a predictive score was devised. Of the 814 patients included, 703 could be evaluated: 164 Res, 266 LA, and 273 Met. The median ages of the patients were 69, 71, and 69, respectively. The median survival times were 21, 15, and nine months, respectively. Six criteria were significantly associated with a lower probability of resectability in multivariate analysis: venous/arterial thrombosis (*p* = 0.017), performance status 1 (*p* = 0.032) or ≥ 2 (*p* = 0.010), pain (*p* = 0.003), weight loss ≥ 8% (*p* = 0.019), topography of the tumor (body/tail) (*p* = 0.005), and maximal tumor size 20–33 mm (*p* < 0.013) or >33 mm (*p* < 0.001). The BACAP score was devised using these criteria with an accuracy of 81.17% and an area under the receive operating characteristic (ROC) curve of 0.82 (95% confidence interval (CI): 0.78; 0.86). The presence of pejorative criteria or a BACAP score < 50% indicates that further investigations and even neoadjuvant treatment might be warranted. Trial registration: NCT02818829.

## 1. Introduction

Pancreatic ductal adenocarcinoma (PDAC) remains a major world health concern as it is the third most common cause of death due to cancer in Western countries. By 2030, it is projected to be the second leading cause of death due to cancer [1,2]. The five-year survival for all stages is very low (7% to 8%) [1,3,4] and surgery is still the only curative treatment, although this is only possible in 15% to 20% of the cases [5]. The poor prognosis for PDAC did not improve over the past several decades even in resectable patients; the mean overall survival is not significantly different for the 1980s (23.2 months), 1990s (25.6 months), and 2000s (24.5 months) [6,7,8].

Of the remaining 80–85% patients who are unresectable due to locally advanced disease, vascular invasion, or metastases, up to 30% are found to be inoperable during explorative laparotomy [9,10]. Recently, in France, the use of preoperative diffusion MRI to detect liver metastases changed the management and frequency of unnecessary laparotomies and pancreatectomies for 10% of the potentially resectable PDAC patients [11]. However, despite improvements in imaging, there is still a need to identify and combine clinico-radiological prognostic factors to help physicians stage the disease early in its course and devise and implement the optimal treatment [12].

Several authors attempted to identify predictive factors of PDAC resectability on diagnosis. Preoperative carbohydrate antigen 19-9 (CA 19-9), the maximal tumor size, and location in the body and tail of the pancreas are the main predictive factors for poor PDAC resectability [13,14,15,16,17,18,19]. However, most of these studies focused on only one or two predictive factors, mainly retrospectively, which resulted in clear biases. Therefore, a large prospective cohort should be established to study a range of predictive factors of PDAC resectability.

To address the lack of clinico-radiological and biological PDAC data, a French multicenter cohort named “BACAP” (the national Anatomo-Clinical Database on Pancreatic Adenocarcinoma), which includes clinical and epidemiological data linked to biological samples of PDAC patients, was developed [20]. This unique large multicenter cohort is a “snapshot” of the current management and outcomes of patients with PDAC in France. The BACAP cohort (universities or private hospitals) has a high level of representativeness of current practices in terms of the diagnosis and treatment of PDAC patients in France.

The aim of this study was to use the data from the BACAP cohort to determine the profile of patients with resectable PDAC and to develop the first predictive scoring system to determine PDAC resectability on diagnosis, using clinico-radiological criteria.

## 2. Results

### 2.1. Patients

**Figure 1:**
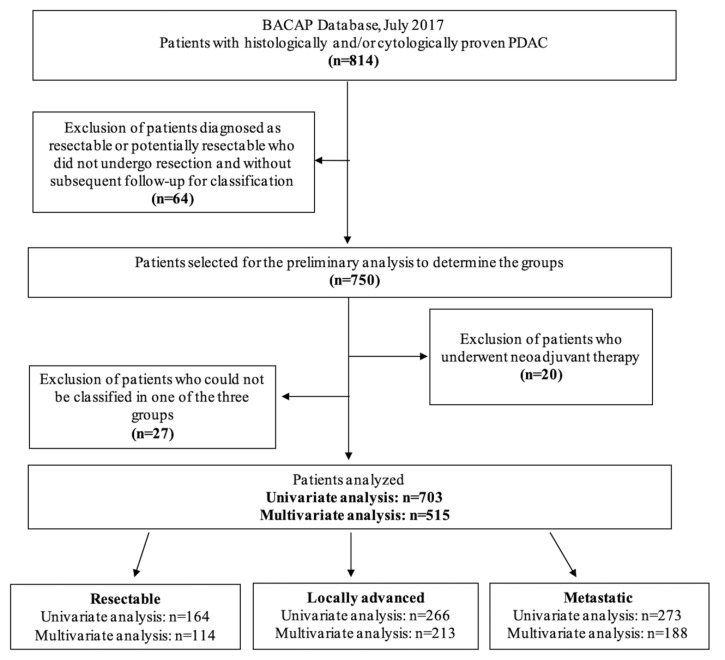
Flowchart. BACAP: National Anatomo-Clinical Database on Pancreatic Adenocarcinoma; PDAC: pancreatic ductal adenocarcinoma.

Between May 2014 and July 2017, 814 patients with histologically and/or cytologically proven PDAC were included in the BACAP cohort. In the univariate analysis, 703 patients were included: 164 patients (23%) in the resectable (Res) group, 266 patients (38%) in the locally advanced (LA) group, and 273 patients (39%) in the metastatic (Met) group. After exclusion of the patients with more than 20% of missing data, 515 patients were included in the multivariate analysis: 114 patients (22%) in the Res group, 213 patients (41%) in the LA group, and 188 patients (37%) in the Met group (Figure 1).

### 2.2. Baseline Characteristics

The baseline characteristics are listed in Table 1 (univariate analysis). The presence of venous or arterial thrombosis, weight loss, abdominal pain, and a WHO (World Health Organization) performance status score ≥ 2, as well as the level of carbohydrate antigen 19-9 (CA 19-9) or carcinoembryonic antigen (CEA) and the maximal tumor size (with higher values in the locally advanced and metastatic groups), were significantly different between the groups (*p* < 0.001).

The presence of jaundice, location of the tumor in the head of the pancreas, and acute pancreatitis on diagnosis were also significantly different between the groups (*p* < 0.001), with a higher frequency in the Res group. In the Res group, pancreatoduodenectomy was performed in 79.4% of the cases. Biliary drainage was performed in 34.3% of the cohort. The main imaging examination to detect PDAC was abdominal CT scan in 75% of the cases, followed by ultrasound endoscopy fine-needle aspiration in 22% of the cases. There were no significant differences between the three groups in terms of the length of time between the onset of symptoms and the first radiological examination (36 days (0–2500) for the Res group, 41 days (0–750) for the LA group, and 37 days (0–404) for the Met group, *p* = 0.304), or between the onset of symptoms and the pathological analysis (48 days (5–2500) for the Res group, 53.5 days (8–765) for the LA group, and 49 days (5–429) for the Met group, *p* = 0.09).

### 2.3. Clinico-Radiological Variables That Predict the Resectability Status

The multivariate analysis identified six clinico-radiological factors that significantly reduced the probability of having a resectable tumor on diagnosis: the presence of venous or arterial thrombosis (*p* = 0.017), a WHO performance status score of 1 vs. 0 (*p* = 0.032) or ≥ 2 (*p* = 0.010) vs. 0, weight loss ≥8% (*p* = 0.019), abdominal pain (*p* = 0.003), location of the tumor in the body or the tail of the pancreas (*p* = 0.005), and a maximal tumor size between 20 and 33 mm (*p* = 0.013) or more than 33 mm vs. ≤20 mm (*p* < 0.001) (Table 2).

The biological data were not included in the multivariate analysis due to missing data, for statistical relevance.

### 2.4. The Predictive Score (the BACAP Score)

Based on these multivariate analysis results, we devised a predictive scoring system to determine PDAC resectability on diagnosis using the six clinico-radiological variables in Table 2. The scoring system is detailed in Table 3, and a website was created to facilitate the use of this score and to enable routine use (http://jdlp.fr/resectability/). Using a cutoff value of 0.5, the model predicted the resectability status in 81.17% of the cases and the area under the receiver operating characteristic (ROC) curve was 0.8205 (95% confidence interval (CI): 0.78; 0.86).

A multivariate logistic model with variables significant at a level of 5% was used to devise the following predictive score (Equation (1)):

(1) $$\mathrm{Probability}\mathrm{of}\mathrm{resectability}(\%)=(\frac{1}{1+\exp\left\{-\left({\hat{\beta }}_{0}+{\hat{\beta }}_{1}x_{1}+{\hat{\beta }}_{2}x_{2}+{\hat{\beta }}_{3}x_{3}+{\hat{\beta }}_{4}x_{4}+{\hat{\beta }}_{5}x_{5}+{\hat{\beta }}_{6}x_{6}\right)\right\}})\times 100$$

where ${\hat{\beta }}_{0}$= 1.28 and ${\hat{\beta }}_{i}$, i = 1, …, *n* are the other estimations from the logistic regression analysis.

### 2.5. Overall Survival

**Figure 2:**
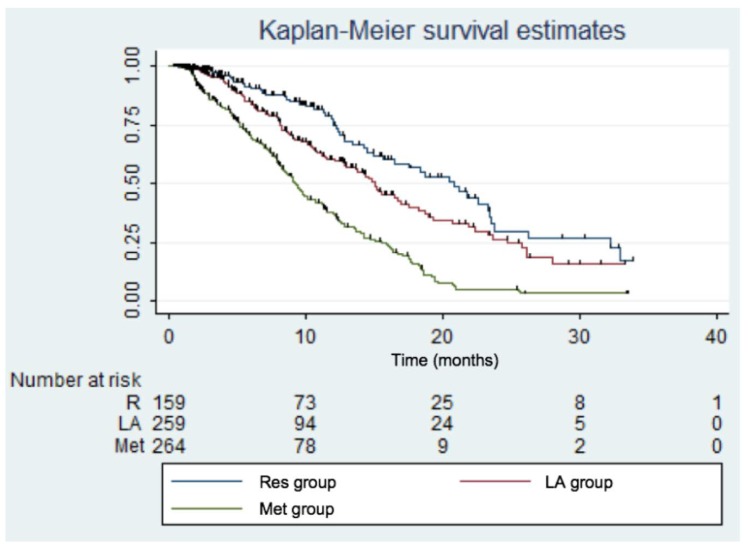
Kaplan–Meier survival estimates according to each group. Res: resectable; LA: locally advanced; Met: metastatic. Log-rank test: *p* < 0.001.

The median follow-up was 12.3 months. The median survival rate was 12.8 months (95% CI: 11.9; 14.3) for all the patients, while it was 20.8 months (95% CI: 15.6; 23.5) for the Res group, 15 months (95% CI: 12.8; 17.5) for the LA group, and 9.2 months (95% CI: 8.2; 10.5) for the Met group. The one-year survival rate was 54.8% (95% CI: 50.0; 59.3) for all the patients and 76.4% (95% CI: 66.6; 83.6) for those in the Res group, 60.5% (95% CI: 52.3; 67.7) for those in the LA group, and 37.5% (95% CI: 30.5; 44.4) for those in the Met group (Figure 2).

**Figure 3:**
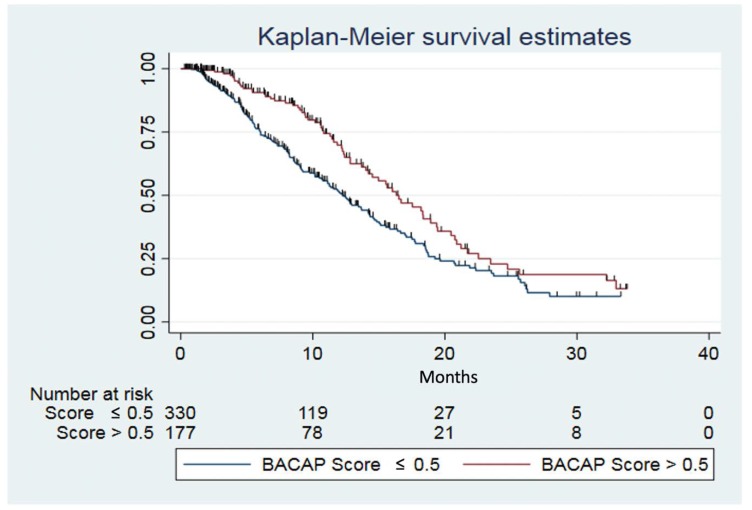
Kaplan–Meier survival estimates according the BACAP score. Log-rank test (*p* = 0.0012).

According to the BACAP score, the median survival rate for the patients with a BACAP score ≤ 0.5 was 12.3 months (95% CI: 10.7; 14.2), while it was 16.3 months (95% CI: 14.0; 18.9) for the patients with a BACAP score > 0.5 (Figure 3). The one-year survival rate for the patients with a BACAP score ≤ 0.5 was 51.4% (95% CI: 44.6; 57.7), while it was 69.8% (95% CI: 60.1; 77.6) for the patients with a BACAP score >0.5.

## 3. Discussion

Despite major advances in PDAC management, this disease remains a challenge for physicians due to the difficulty of early diagnosis. Among the various PDAC classifications in the literature [21], the most commonly used is the National Comprehensive Cancer Network (NCCN) system which classifies PDAC patients into three groups: resectable (including borderline resectable PDAC), locally advanced, and metastatic [22]. Vascular involvement of more or less than 180 degrees (with portomesenteric confluence, hepatic artery, celiac artery, or inferior vena cava) is the main factor that allows differentiation of the resectable group (including borderline tumors) from the locally advanced group [22]. The shortcoming of this classification is that it is only based on morphological criteria and does not include biological or clinical criteria, which leads to unnecessary laparotomies in as many as 30% of the PDAC patients considered to be resectable [9,10]. Therefore, we tried to determine the clinico-radiological predictive factors of resectability using the largest prospective multicentric cohort of PDAC patients to date with clinico-radiological and biological data described in the literature: the BACAP cohort.

Up until now, most studies that investigated the predictive factors of resectability were retrospective and monocentric, and they only assessed one or two criteria. In this study, we identified in multivariate analysis six clinico-radiological criteria that significantly reduced the probability of having a resectable tumor on diagnosis. These factors are well known to clinicians and are widely described in the literature; a large tumor size [18,23,24,25], location of the tumor in the body or tail [19,26,27,28,29], abdominal pain [30,31], weight loss ≥ 5–10% [32,33], venous or arterial thrombosis [34,35], and a poor performance status score (PS) [36,37,38] correlate with unresectability and poor survival of PDAC patients.

In the literature, only a few studies developed a score to predict resectability. These studies were monocentric, with a limited population, were mainly retrospective, and only used radiological criteria [39,40,41]. To date, only one study associated radiological criteria with tumor biomarkers to extend the basis of a predictive score [41]. Based on the multivariate analysis, we developed the first predictive scoring system to determine PDAC resectability, using both clinical and radiological criteria. This BACAP score allowed us to consider the patient as a whole and not limit ourselves to morphological criteria only. The main strengths of this score are its high efficiency and its ease of use with only six clinico-radiological criteria. For example, on diagnosis, a patient with no venous or arterial thrombosis, a performance status score of one, with abdominal pain, weight loss ≥ 8%, and a tumor ≤ 20 mm located in the head of the pancreas has a 35% probability of being resectable. We would like to emphasize the importance of having such a tool in clinical practice to assist with the therapeutic decision in the context of a multidisciplinary meeting. However, this score still needs to be validated at the international level.

The main limitation of this study is the large amount of missing biological data. Therefore, Ca 19-9 and CEA were significant in univariate analysis but were no longer significant in multivariate analysis. Thus, these two biomarkers were not included in the BACAP score, although numerous authors showed that these biomarkers correlate with the prognosis and advanced stage disease [3,17,42,43].

## 4. Materials and Methods

### 4.1. Population

Fifteen hospitals in France currently contribute data to the French BACAP prospective cohort. This biobank includes clinical, radiological, epidemiological, and social data linked to the biological samples. Patients with any stage of histologically and/or cytologically proven pancreatic ductal adenocarcinoma were included in the BACAP cohort on diagnosis and before treatment from May 2014 to July 2017. The design and management of the BACAP cohort were described in a previous article [20].

The patients were divided into three groups: the resectable (Res) group, the locally advanced (LA) group, and the metastatic (Met) group. The Res group included the patients for whom all the paraclinical examinations led to the conclusion of “resectable tumor” or “potentially resectable tumor” and who actually underwent surgery. The LA group included the patients for whom all the paraclinical examinations led to the conclusion of “locally advanced tumor”. In this group, the CT scan or exploratory laparotomy revealed stomach, duodenal, colon, superior mesenteric artery, hepatic artery, coeliac trunk, superior mesenteric vein, portal vein, and splenic vein invasion. The Met group included the patients for whom all the paraclinical examinations led to the conclusion of “metastatic tumor”. Liver metastasis or peritoneal carcinomatosis could be diagnosed during laparoscopy or laparotomy.

### 4.2. Main Objective

The main objective of this study was to develop a predictive score (the BACAP score) from clinical and radiological criteria to determine the probability of having a resectable PDAC on diagnosis.

### 4.3. Data Collected

For each patient, variables were collected prospectively and are detailed in a supplementary data table (Table S1). The initial data collected at inclusion were (1) sociodemographic data, (2) medical history, (3) symptoms on diagnosis, (4) biliary drainage, (5) description and characteristics of the pancreatic disease and the tumor, (6) biological analysis on diagnosis, (7) anatomopathological analysis, (8) treatment, and (9) delays. The WHO performance status score measures cancer patients’ degree of autonomy: 0 (asymptomatic, fully active), 1 (restricted in physically activity but able to do everything else), and 2 (unable to work, out of bed more than 50% of the time during the day).

### 4.4. Statistical Methods

The population analyzed included the eligible patients who were previously classified in one of the three groups: resectable, locally advanced, or metastatic. Descriptive statistics were done of the entire population that was analyzed and in each group. The quantitative variables were described by the median and the range (minimum-maximum). The qualitative variables were described by the number of observations and the proportions. The number of missing values was reported, and the proportions calculated excluding missing values. The Kruskal–Wallis test was used to compare the distribution of the quantitative variables between the three groups. Proportions were compared between the three groups using chi-squared tests or Fisher’s exact test when observations were at a low limit (expected cell count less than five). The variables that were significant at a level of 5% (*p* ≤ 0.05) in the univariate analysis were selected for the multivariate analysis. To accomplish the main objective, the probability of belonging to the Res group vs. the LA or the Met group was calculated by multinomial logistic regression modeling. A multivariate logistic model with variables significant at a level of 5% was used to devise the predictive score. We checked goodness of fit using the Hosmer–Lemeshow test and performed a link test to detect potential errors of model specification. The efficiency of the score was assessed by calculating the rate of correctly classified patients and the area under the receiver operating characteristic curve (ROC curve). The survival curves and rates were estimated with the Kaplan–Meier method, and the overall survival rates between the groups were compared using a log-rank test. All the statistical tests were bilateral with a significance level of 5%. The statistical analyses were performed with STATA v13.0 (StataCorp LP, College Station, TX, USA).

### 4.5. Ethics Statement and Consent to Participate

All the patients were informed of the study and voluntarily consented in writing to being added to the BACAP cohort (Biological and Clinical Database for Pancreatic Adenocarcinoma). The biological collection was declared to and approved by the French Ministry of Research under number DC-2013-1974 and the database was mentioned in Clinical Trials under the following number: NCT02818829.

## 5. Conclusions

This study conducted in a large multicentric prospective cohort evaluated current management and outcomes of PDAC in France. We found six predictive clinico-radiological criteria of resectability that allowed us to devise a resectability scoring system that can be used on diagnosis. The BACAP score can be readily generated and will assist physicians with routine prediction of the resectability status of PDAC patients on diagnosis using straightforward criteria. The presence of adverse criteria or a BACAP score < 50% indicates that further investigations (such as MRI of the liver and of the pancreas, endoscopic ultrasound, repeat CT scan, etc.) are warranted to diagnose the PDAC more precisely, which could reduce the number of unwanted laparotomies. This would allow the patients to receive the best treatment or even a neoadjuvant treatment prior to resection.

## Figures and Tables

**Table 1:** PDAC patient characteristics at baseline (univariate analysis).

PDAC Patient Characteristics at Baseline (Univariate Analysis)	Res (*n* = 164)	LA (*n* = 266)	Met (*n* = 273)	Total (*n* = 703)	*p*
***Demographic Data***					
Age on diagnosis (median, range) (MD)	69 (21–88) (0)	71 (34–91) (0)	69 (36–91) (0)	70 (21–91) (0)	0.070
Gender (n, %) (MD)					0.408
Female	73 (44.5) (0)	129 (48.5) (0)	117 (42.9) (0)	319 (45.4) (0)	
Male	91 (55.5) (0)	137 (51.5) (0)	156 (57.1) (0)	384 (54.6) (0)	
Body mass index on diagnosis (median, range) (MD)	24.4 (15–52) (3)	23.5 (15.6–114.3) (5)	24.1 (14.2–112) (13)	23.9 (14.2–114.3) (21)	0.05
**Medical History**					
WHO performance status (n, %) (MD)					< 0.001
0	76 (58.9) (35)	86 (36.3) (29)	88 (37.1) (36)	250 (41.5) (100)	
1	48 (37.2) (35)	124 (52.3) (29)	101 (42.6) (36)	273 (45.3) (100)	
≥2	5 (3.9) (35)	27 (11.4) (29)	48 (20.3) (36)	80 (13.3) (100)	
Smoking history (n, %) (MD)					0.097
Non-smoker	76 (46.3) (0)	145 (54.9) (2)	131 (49.1) (6)	352 (50.7) (8)	
Former smoker	46 (28.1) (0)	75 (28.4) (2)	89 (33.3) (6)	210 (30.2) (8)	
Current smoker	42 (25.6) (0)	44 (16.7) (2)	47 (17.6) (6)	133 (19.1) (8)	
Alcohol consumption (n, %) (MD)					0.791
Non-consumer	100 (61.4) (1)	174 (66.4) (4)	179 (66.5) (4)	453 (65.3) (9)	
Former consumer	17 (10.4) (1)	21 (8.0) (4)	24 (8.9) (4)	62 (8.9) (9)	
Current consumer	46 (28.2) (1)	67 (25.6) (4)	66 (24.5) (4)	179 (25.8) (9)	
Family history of cancer (n, %) (MD)	66 (40.2) (0)	123 (46.2) (0)	128 (47.1) (1)	317 (45.2) (1)	0.346
Diabetes (n, %) (MD)	44 (26.8) (0)	74 (27.8) (0)	61 (22.4) (1)	179 (25.5) (1)	0.323
Other pancreatic diseases * (n, %) (MD)	30 (18.3) (0)	20 (7.5) (0)	15 (5.5) (0)	65 (9.3) (0)	< 0.001
**Clinical condition on diagnosis**					
Venous or arterial thrombosis (n, %) (MD)	7 (4.3) (0)	39 (14.7) (0)	51 (18.7) (0)	97 (13.8) (0)	< 0.001
Weight loss (n, %) (MD)	90 (55.6) (2)	200 (76.1) (3)	186 (68.6) (2)	476 (68.4) (7)	< 0.001
Abdominal pain (n, %) (MD)	89 (54.3) (0)	193 (72.8) (1)	201 (73.9) (1)	483 (69) (2)	< 0.001
Jaundice (n, %) (MD)	80 (48.8) (0)	116 (43.8) (1)	68 (25) (1)	264 (37.7) (2)	< 0.001
Acute pancreatitis (n, %) (MD)	19 (11.6) (0)	11 (4.2) (2)	8 (2.9) (1)	38 (5.4) (3)	< 0.001
Tumor Characteristics and Procedures					
Maximal tumor size (mm) (median, range) (MD)	27 (7–76) (15)	35 (4–85) (10)	36 (2–150) (12)	33 (2–150) (37)	< 0.001
Tumor location: head of pancreas (n, %) (MD)	117 (72.2) (2)	169 (63.8) (1)	125 (47) (7)	411 (59.3) (10)	< 0.001
Biliary drainage (%) (MD)	60 (36.6) (0)	112 (42.1) (0)	69 (25.3) (0)	241 (34.3) (0)	< 0.001
Surgical resection by pancreaticoduodenectomy (n, %) (MD)	58 (79.4) (0)	0 (0)	0 (0)	58 (8.3) (0)	-
**Biology on Diagnosis**					
Serum bilirubin (μmol/L) (median, range) (MD)	32.5 (2.5–533.3) (34)	32 (3.4–647) (83)	12.8 (3.4–548) (78)	18.4 (2.5–647) (195)	0.004
CA 19-9 (IU/mL) (median, range) (MD)	183.5 (0.1–5314) (84)	247.6 (0–240000) (152)	521.7 (0–135720) (129)	261.2 (0–240000) (365)	< 0.001
CEA (IU/ml) (median, range) (MD)	3 (0.6–44.4) (99)	5 (0.5–3862) (177)	11 (0.7–11394) (147)	5.3 (0.5–11394) (423)	< 0.001

Res: Resectable; LA: locally advanced; Met: metastatic; PDAC: pancreatic ductal adenocarcinoma; MD: missing data; CEA: carcinoembryonic antigen; CA 19-9: carbohydrate antigen 19-9; weight variation (%): (weight on diagnosis − usual weight)/usual weight × 100. * Intraductal papillary mucinous tumor of the pancreas, mucinous cystadenoma, serous cystadenoma, chronic pancreatitis, and/or hereditary pancreatitis.

**Table 2:** Predictive clinico-radiological factors of resectability (multivariate analysis).

Predictive Clinico-Radiological Factors of Resectability (Multivariate Analysis)	OR	95% CI	*p*
Venous or arterial thrombosis on diagnosis			
No			
Yes	0.30	0.11; 0.81	0.017
WHO Performance Status on diagnosis			
0			
1	0.58	0.35; 0.95	0.032
≥ 2	0.25	0.09; 0.72	0.010
Weight loss on diagnosis			
≤8%			
≥ 8%	0.55	0.34; 0.91	0.019
Abdominal pain on diagnosis			
No			
Yes	0.46	0.28; 0.76	0.003
Location of the tumor			
Head			
Body and tail	0.42	0.24; 0.75	0.005
Maximal tumor size (mm)			
≤20			
20–33	0.49	0.28; 0.86	0.013
> 33	0.11	0.05; 0.20	<0.001

OR: odds ratio; CI: confidence interval (*n* = 515).

**Table 3:** The BACAP score: the scoring system to determine the resectability of pancreatic ductal adenocarcinoma. Exp: exponential function.

The BACAP Score	𝑥_n_	𝛽_i_
Venous or arterial thrombosis on diagnosis: $x_{1}$		
No	0	
Yes	1	−1.21
WHO Performance Status on diagnosis: $x_{2}$		
0	0	
1	1	−0.55
≥2	2	−1.37
Weight loss on diagnosis: $x_{3}$		
<8% on diagnosis	0	
≥8% on diagnosis	1	−0.59
Abdominal pain on diagnosis: $x_{4}$		
No	0	
Yes	1	−0.77
Location of the tumor: $x_{5}$		
Head	0	
Body and tail	1	−0.86
Maximal tumor size: $x_{6}$		
≤20 mm	0	
20–33 mm	1	−0.70
>33 mm	2	−2.25

## Supplementary Materials

The following are available online at https://www.mdpi.com/2072-6694/12/4/783/s1: Table S1: Clinico-radiological and biological data collected.

## Abbreviations

ALT	alanine aminotransferase
AST	aspartate aminotransferase
BACAP	National Anatomo-Clinical Database on Pancreatic Adenocarcinoma
CA19-9	Carbohydrate antigen 19-9
CEA	Carcinoembryonic antigen
CT-scan	Computed tomography scan
EUS	Endoscopic ultrasound
ERCP	Endoscopic retrograde cholangiopancreatography
INR	International Normalized Ratio
IPMN	Intraductal papillary mucinous neoplasm
LA	Locally advanced
Met	Metastatic
MRI	Magnetic resonance imaging
MTM	Multidisciplinary Team Meeting
PDAC	Pancreatic ductal adenocarcinoma
PET/CT	Positron emission tomography/computed tomography
PS	Performance status
Res	Resectable
ROC curve:	Receiver operating characteristic curve
TNM	Tumor node metastasis
WHO	World Health Organization
