# Supplementary material for: A New Score to Predict the Resectability of Pancreatic Adenocarcinoma: The BACAP Score
Source: Cancers (Basel). 2020 Mar 25;12(4):783. doi: 10.3390/cancers12040783 (PMC7226323; doi:10.3390/cancers12040783)
Supplement: Supplementary file 1 [file cancers-12-00783-s001.pdf]

Supplementary Materials: A New Score to Predict the Resectability of Pancreatic Adenocarcinoma: The BACAP Score

Charlotte Maulat, Cindy Canivet, Célia Touraine, Sophie Gourgou, Bertrand Napoleon, Laurent Palazzo, Nicolas Flori, Guillaume Piessen, Pierre Guibert, Stéphanie Truant, Eric Assenat, Louis Buscail, Barbara Bournet and Fabrice Muscari

Table S1. Clinico-radiological and biological data collected.

	**Socio-demographic data**	
**Gender**	**Educational level**	**Occupation**
**Male**	No diploma	Farmer
**Female**	General Certificate of Education	Artisan, storekeeper
**Age**	Vocational diploma	Executive, higher education profession
	Vocational training certificate	Intermediate level occupation
	Baccalaureate (SAT)	Employee
	Baccalaureate (SAT) +2	Manual worker
	Educational certificate	Retired
	Other	No professional activity
		Other
	**Medical history**	
**Smoking history**	**Alcohol consumption**	**Family history of cancer**
**Non-smoker**	Non consumer	Yes
**Former smoker**	Former consumer	No
**Current smoker**	Current consumer	
**Other pancreatic diseases**	**History of diabetes**	
**Yes**	Yes	
**No**	No	
	**Symptoms on diagnosis**	
**Abdominal pain**	**Jaundice**	**Chronic pancreatitis**
**Yes**	Yes	Yes
**No**	No	No
**Random**	**Weight loss**	**Recent diabetes**
**Yes**	Yes	Yes
**No**	No	No
**Anorexia**	**Acute pancreatitis**	*Gastrointestinal hemorrhage*
**Yes**	Yes	Yes
**No**	No	No
**Biological anomaly**	**Upper gastrointestinal obstruction**	**Arterial or venous thrombosis**
**Yes**	Yes	Yes
**No**	No	No
**Ascites**	**Transit disorders**	**Cholestatic syndrome**
**Yes**	Yes	Yes
**No**	No	No
**Cholecystitis**	**Fever**	**WHO performance status**
**Yes**	Yes	0
**No**	No	1
**Usual BMI**	**Current weight**	2
**Current BMI**	**Usual weight**	3
	**Biliary drainage**	
**Biliary drainage**	**Percutaneous drainage**	**Endoscopic drain**

**Yes**	Yes	Metallic
**No**	No	Plastic
ERCP	**Radiological drain**	Drain
**Yes**	Metallic	
**No**	Plastic	
	Drain	
**Description and characteristics of the pancreatic disease and the tumor (CT Scan or Endoscopic ultrasound)**		
**Location of the tumor**	**Maximal tumor size (mm)**	**Type of initial imaging**
**Head**	**Conclusion of the paraclinical examinations**	Chest CT scan
**Isthmus**	Resectable	Abdominal CT scan
**Body**	Potentially resectable	Endoscopic ultrasound
**Tail**	Locally advanced	**IPMN**
**Multiple**	Metastatic	Yes
		No
**Biological analysis on diagnosis**		
**Hemoglobin (g/dl)**	INR	Gamma-GT (IU/l)
**Leucocytes (G/l)**	Albumin (g/l)	Alkaline Phosphatase (IU/l)
**Neutrophils (G/l)**	AST (IU/l)	Total bilirubin (µmol/l)
**Platelets (G/l)**	ALT (IU/l)	Creatinine (µmol/l)
**CA 19-9 (IU/ml)**	CEA (ng/ml)	
**Anatomopathological analysis**		
**Puncture site**	**Immunocytochemistry**	**Immunohistochemistry**
**Primary tumor**	Yes	Yes
**Liver**	No	No
**Node**		
**Another metastasis**		
**Multiple**		
**Treatment**		
**Resectable group treatment (surgery and after surgery)**		
**Type of surgery**	**TNM staging**	**Clavien-Dindo classification**
**Whipple procedure**	Ia	Grade I
**Left pancreatectomy without splenic preservation**	Ib	Grade II
**Left pancreatectomy with splenic preservation**	IIa	Grade IIIa
**Pancreatic fistula**	IIb	Grade IIIb
**Yes**	III	Grade IVa
**No**	IV	Grade IVb
		Grade V
**Locally advanced group treatment (type of chemotherapy)**		
**Metastatic group treatment (type of chemotherapy)**		
**Delays (days)**		
**Between symptoms and first radiological exam**	Between biopsy and surgery	Between biopsy and first line chemotherapy
**Between symptoms and anatomopathological analysis**	Between symptoms and first line chemotherapy	Between MTM inclusion and surgery
**Between symptoms and surgery**	Between MTM inclusion and first line chemotherapy	Between first line of chemotherapy and tumor progress

BMI: Body Mass Index, IPMN: intraductal papillary mucinous neoplasm, ERCP: Endoscopic Retrograde Cholangiopancreatography, CT scan: Computed Tomography scan, IPMN: intraductal papillary mucinous neoplasm, MTM: Multidisciplinary Team Meeting, WHO: World Health Organization, AST: Aspartate aminotransferase, ALT: Alanine aminotransferase, CEA: Carcinoembryonic antigen, Ca 19-9: Carbohydrate antigen 19-9.
